# A cost-effectiveness analysis of melatonin in comparison with midazolam for anxiety prior to general anaesthesia in children: the MAGIC randomised controlled trial (melatonin for anxiety prior to general anaesthesia in children)

**DOI:** 10.1186/s12871-025-03489-x

**Published:** 2025-12-17

**Authors:** Tracey A. Young, Marie E Hyslop, Diana E Papaioannou, Christopher Deery, Robert Bolt, Matthew J Wilson, Christopher Vernazza, Esther Herbert, Nikki Totton, Laura Flight, MAGIC Collaborative, Janet Clarkson, Christopher Evans, Nicholas Ireland, Jennifer Kettle, Zoe Marshman, Amy C Norrington, Robert H Paton, Sondos Albadri, Laura Armstrong, Simon Atkins, Margaret Babb, Claire Biercamp, Katie Biggs, Mike Bradburn, Jaimie Buckley, Julie Child-Cavill, Sean Cope, Simon Crawley, Munya Dimairo, Enass Duro, Ayman Eissa, Jacqui Gath, Gil Gavel, Tim Geary, Fiona Gilchrist, Padma Gopal, Jamie Hall, Kate Hutchence, Puran Khandelwal, Pranav Kukreja, Ian Leeuwenberg, James Limb, Amanda Loban, Katie Mellor, Nuria Masip, Anthony Moores, Vimmi Oshan, Edward Pickles, Jaydip Ray, Helen Rodd, Sian Rolfe, Elena Sheldon, Richard Simmonds, Rachel Smith, Ashok Sundar, Anna Thomason, Simon Waterhouse, Graham Wilson, Julian Yates

**Affiliations:** 1 Sheffield Centre for Health and Related Research (SCHARR), School of Medicine and Population Health, University of Sheffield, Sheffield, S1 4DA UK; 2 Clinical Trials Research Unit, Sheffield Centre for Health and Related Research (SCHARR), School of Medicine and Population Health, University of Sheffield, Sheffield, S1 4DA UK; 3 School of Clinical Dentistry, University of Sheffield, Sheffield, S10 2TA UK; 4 School of Dental Science, Newcastle University, Newcastle-Upon-Tyne, NE2 4BW UK

**Keywords:** Cost-effectiveness, Premedication, Paediatric

## Abstract

**Background:**

To date no study has looked at the cost-effectiveness of melatonin for anxiety prior to general anaesthetic in children or young people. The aim of the health economic analysis was to evaluate the within trial cost-effectiveness of melatonin for anxiety in children compared to usual care (midazolam) prior to general anaesthesia in children from an NHS and Personal Social Services perspective.

**Methods:**

The economic evaluation was undertaken alongside a multicentre randomised controlled trial (MAGIC). Children were individually randomised to receive either melatonin or midazolam for anxiety prior to general anaesthesia. Resource use was collected from case-record forms. Children were followed up at 14 days post-surgery. The main outcome was the incremental cost per successful procedure. The trial was closed early due to recruitment futility, which limited the studies statistical power.

**Results:**

A total of 100 children received the Investigational Medicinal Product (IMP) treatment, 50 receiving melatonin and 50 receiving midazolam, these were the focus of the health economic analysis. On average, costs over 14 days were lower for those who received melatonin (-£46.20, 95% CI: -£166.14 to £66.74) with a mean incremental difference in procedure success of -0.02 (95% CI –0.08 to 0.004), though there was uncertainty around the results. There was no evidence of either treatment being cost-effective in a cost per QALY analysis using the CHU-9D (-£46.20, 95% CI: -£166.142 to £66.74) with a mean incremental QALY -0.0001 (95% CI –0.0008 to 0.0008). Subgroup analysis was limited to those who underwent head and neck procedures owing to small numbers by subgroup for other procedure types and age group and results were similar to the main analysis.

**Conclusions:**

This is the first study to examine the cost-effectiveness of melatonin in comparison with midazolam in children. The results were inconclusive showing no evidence that melatonin was more cost-effective than midazolam. The study closed early owing to issues with recruitment, which reduced the studies statistical power, and this has limited the economic analysis.

**Trial registration:**

Registered with the UK Clinical Study Registry ISRCTN18296119 on 10/01/2019.

**Supplementary Information:**

The online version contains supplementary material available at 10.1186/s12871-025-03489-x.

## Background

Midazolam is the current first choice premedication for an anxious child requiring a general anaesthetic [1]. Midazolam is effective, although it has potential side effects including loss of coordination and risks to breathing [2]. Midazolam can also have unpredictable effects on anxiety, with some children becoming over excited rather than being calmed [3]. Midazolam has amnesic properties which can be an advantage and a disadvantage depending on the situation [4]. Melatonin, which also has anxiolytic properties, offers an alternative premedication, and has shown promise as it avoids midazolam’s side effects [5]. However, the relatively small number of previous trials of melatonin as a premedication in children have generally involved small samples and have been drawn from the general surgical population rather than focusing on anxious patients, therefore diluting the identification any anxiolytic effect on these patients who require premedication. A recent systematic review of the use of melatonin as a premedication concluded there was not adequate evidence to confirm that melatonin is as effective as current premeditations [5].

There have been a few studies that have looked at the cost-effectiveness of midazolam [6–8] but to date no study has examined the cost-effectiveness of melatonin compared with midazolam in children and young people. This paper presents the within trial results of the cost effectiveness analysis of melatonin compared to midazolam in anxious children for day-case elective ENT, ophthalmological, dental, gastroenterology, radiology, plastic, orthopaedic, urology and other general surgery using data from the Melatonin for Anxiety prior to General anaesthesia in Children (MAGIC) randomised controlled trial. The cost-effectiveness study examines results over the study period using both a cost per successful procedure and cost-per QALY approach from an NHS and Personal Social Services perspective.

## Methods

### Study design and population

Full details of the study design and trial results are described elsewhere [9, 10]. The study was a parallel group, double blind, randomised controlled trial (RCT) to evaluate the non-inferiority of melatonin against midazolam in dealing with pre-operative anxiety in children undergoing day-case elective surgery. The study recruited children between the ages of 3 and 14 years who were undergoing day-case elective surgery for: elective dental, ophthalmological, ENT, gastroenterology, radiology, plastic, orthopaedic, urology or other general surgery under general anaesthesia. Children were recruited across 20 UK hospital trusts and were identified for day-case elective surgery as per local standard care. Parental/carer’s consent was obtained for inclusion in the study. Children were followed up to 14 days post-surgery, information for the trial was collected during the hospital visit and at 14 days post-surgery. The primary outcome measure in the trial was the modified Yale Preoperative Anxiety Scale-Short Form (mYPAS-SF) which showed anxiety to be significantly less for those in the midazolam arm [10], this difference was also clinically meaningful [11]. The trial closed early due to recruitment futility with 110 recruited of the target of 624, therefore there was an increased risk of bias. The only difference between the arms identified was a chance imbalance in baseline mYPAS-SF scores in favour of midazolam, the analysis accounted for any potential bias this difference may have introduced by looking at adjusted scores rather than raw scores.

### The treatment arms

Both midazolam and melatonin were administered at 0.5 mg/kg dose, with a capped dose of 20 mg in 20 ml. Children received a single dose of either midazolam or melatonin on the day of surgery approximately 30 min prior to transfer to theatre. The main trial [10] reported that there were no serious adverse events (SAE) and of the adverse events (AEs) only one was possibly due to midazolam (agitation).

This trial was registered with the International Standard Randomised Controlled Trial Registry (ISRCTN18296119) and was approved by Liverpool Central Research Ethics Committee (18/NW/0758) and received Medicines and Healthcare products Regulatory Agency (MHRA) approval (21,304/0267/001–0001).

A health economics analysis plan (HEAP) (Supplementary Material 1—SM1) was written and approved by the Trial Steering Committee before the analysis stage. All health economic analysis was conducted in R version 2022.07.0 [12]. Analyses are reported using the Consolidated Health Economic Evaluation Reporting Standards (CHEERS) [13] checklist (SM2), the statistical analysis for the original trial was reported elsewhere [9, 10] in accordance with CONSORT guidelines for pragmatic and noninferiority trials [14, 15] (SM3).

### Outcomes

The primary economic outcome measure was the proportion of successful procedures over the study duration and was defined by surgery not being abandoned before the point of unconsciousness. The secondary economic outcome measure was Quality Adjusted Life Years (QALYs), this was selected as a secondary rather than primary outcome as it was unclear whether sedation has long-term effects on quality of life [9]. QALYs combine both the quality and quantity of life and is measured using utility [16]. Utilities in the MAGIC trial were derived from utility scores obtained using the Child Health Utility 9D (CHU-9D) questionnaire which was administered at baseline and 14 days post-surgery [17]. Measured domains include worried, sad, pain, tired, annoyed, schoolwork/homework, sleep, daily routine and activities. There are five response options per domain. For children under seven years old, a proxy (parent or guardian) completed the questionnaire. It is recognised that proxies tend to report lower quality of life than self-completion (see for example Khanna et al. [18]). Therefore, two cost-utility analyses were carried out. The first did not distinguish between proxy and self-completed responses. In the second analysis only the self-completed responses (children over seven years old) were included. Given the short time frame for the within trial analysis it may only show small health benefits, the third analysis was a cost minimisation analysis which focused on the costs of the interventions and assumed that the effectiveness was equivalent.

### Resource use and unit costs

Resource use linked to the hospital procedure were collected via study case report forms (CRF) during hospitalisation and at 14 days post-surgery. The unit costs of melatonin, midazolam and concomitant medications collected through the CRF were obtained from the British National Formulary for Children (BNFC) [19]. Where multiple supplier costs were available an average was taken across all suppliers. Cost of procedures and out-patient appointments were obtained from the National Cost Collection [20] and GP costs were obtained from the Personal Social Service Research Unit (PSSRU) [21].

Costs are presented using 2021/22 prices. Table 1 summarises the unit cost sources.

**Table 1:** Sources of resource use costs

Services/Medication Used	Source of costs
IMP	
Melatonin, Midazolam	British National Formulary for Children (Joint Formulary Committee 2022)
Procedure	
Primary tooth extraction, permanent tooth extraction, surgical extraction, tonsillectomy, grommets, excision and drainage, squint surgery, adenoidectomy, adenotonsillectomy, minor dental procedure, endoscopy, minor oculoplastic procedure, tympanoplasty, mastoid procedure, minor ocular procedure, deviated septum, ingrowing toenail, circumcision, maxillofacial	National Cost Collection 2021/22
Concomitant medications (during procedure and up to 14 days post procedure)	
Paracetamol, Ibuprofen. Ondansetron, Fentanyl. Dexamethasone, Propofol, Diclofenac, Proxymetacaine, Hartmanns, Morphine, Atracurium, Clondine, Procyclidine, Glycopyrrolate, Ketoralac, Alfentanil, Co-amoxiclav, Plasma-lyte, Renifentanil, Sevoflurane, Amoxicillin, Atrpine, Chloramphenicol, Chlorphenamine, Cyclizine, Diprivan, EMLA cream, Ketamine, Mivacurium, Neostigmine, Nocuron, Nubila, Piriton, Proxymethocaine, Rocuronium, Thiopental, Tranexamic Acid, Vecuronium, Clenil, Salbutamol, Maxidex, Lansoprazole, Naseptin, Atropine Sulphate, Calcium, Calpol, Cetirizine, Chlorhexidine glucomate, Cyclosporin, Difflam, Dulcolax, Equasym, Erythromycin, Flurometholone, Laxido, Mometasone, Monteleukast, Oramorph, Pizotifin, Sodium hyaluronate, Ventolin	British National Formulary for Children (Joint Formulary Committee 2022)
Primary and community services	
Outpatient appointment	National Cost Collection 2021/22
GP	PSSRU 2022

### Cost-effectiveness analysis

The cost-effectiveness analysis deviates from the health economic analysis plan (HEAP) which described a decision analytic model to explore the cost-effectiveness of melatonin over a 1-year time frame (SM1). When this analysis was outlined, it was stated that the analysis would be undertaken “if there is the potential for the cost-effectiveness of melatonin to improve under a longer analysis time horizon than the 14-day follow-up.” After examining the results from the statistical analysis [10] and the within-trial cost-effectiveness results a decision was made not to carry out the modelling analysis as there was no potential for the results to be cost-effective in favour of melatonin over a longer period. The cost-effectiveness analysis presented here gives the results of the within trial analysis over 14 days.

There were no missing resource use data, or primary outcome data. However, 43% of QALY data was missing, 46% in the melatonin arm and 40% in the midazolam arm. Data were assumed to be missing at random as there is a possibility that missingness is related to age of the participant. Missing data were imputed by treatment arm using chained equations to create 100 imputations, information on overall costs, sex, age and surgery type were used to inform the imputations [22]. The fraction of missing information (FMI) was used to inform the number of imputations, this suggested a minimum of 50 imputations were needed. However, given the small sample size to obtain more precise estimates, 100 imputations were implemented.

Confidence intervals around mean costs and QALYs were estimated using bootstrapping, a total of 5000 bootstrap replicates were run. Results are presented on the cost-effectiveness plane and with cost-effectiveness acceptability curves. Incremental cost-effectiveness ratios (ICER) are not presented owing to small health benefits resulting in numerically unstable ICERs. No discounting was applied as the period for the analysis was less than one year.

### Cost-effectiveness from a wider than NHS perspective and subgroup analysis

An NHS and wider perspective was examined using information provided by primary care givers in a cost questionnaire that asked them about how they travelled to their child’s appointment, the distance and time taken to travel and what they would have been doing had they not been at the appointment (lost time/earnings). The cost of car journeys was taken from the UK governments suggested expenses claim for travel of 0.45p per mile [23] and multiplied by distance travelled to obtain a total cost for the journey. The cost of a bus journey was assumed to be £2 per journey as applied under the government bus fare cap [24] and a journey by taxi fare was taken from the taxi-calculator website and based on a base fee of £2.50 plus a rate of £2.70 per mile [25]. Hourly rates of hours and earnings were taken from the Office for National Statistics (ONS) Earnings and Working Hours Survey for 2022 [26]. It was assumed that any time away from usual activities was valued the same for those in or not in paid employment and the average hourly rate for the time spent at the appointment was applied to all participants.

The variability in costs for two sub-groups were explored 1) for surgery specialty (head and neck, gastro and MRI, other) and 2) age (< 7 years, ≥ 7 years). Any subgroups with fewer than 30 participants are reported using descriptive statistics only.

## Results

A total of 110 children were recruited into the study with 55 (50%) randomised to melatonin and 55 (50%) to midazolam. A total of 100 (90.9%) children were administered the Investigational Medicinal Product (IMP) treatment, 50 (90.9%) administered to receive melatonin and 50 (90.9%) administered to receive midazolam. The numbers differ from the clinical results as one child was administered melatonin and the dose was prepared but not administered, there is a pharmacy cost to prepare the dose and therefore this patient was included in the health economic analysis. The average age of children in the economic evaluation was 8 years old and 53% were female. The most common procedure was primary tooth extraction, which almost half of participants underwent (49%), followed by squint surgery (11%) and surgical extraction (7%) (Table 2).

**Table 2:** Summary of procedures undertaken

	Melatonin (*N* = 50)	Midazolam (*N* = 50)	Total (*N* = 100)
Primary tooth extraction	23 (46%)	26 (52%)	49 (49%)
Permanent tooth extraction	2 (4%)	3 (6%)	5 (5%)
Surgical extraction	3 (6%)	4 (8%)	7 (7%)
Tonsillectomy	0 (0%)	2 (4%)	2 (2%)
Grommets	1 (2%)	1 (2%)	2 (2%)
Excision & drainage	0 (0%)	2 (4%)	2 (2%)
Squint surgery	9 (18%)	2 (4%)	11 (11%)
Other procedure	12 (24%)	10 (20%)	22 (22%)

### Successful procedures

A total of three procedures were incomplete (unsuccessful) during the trial. The reasons were 1 spat out the medication, 1 was too nervous, 1 the procedure was abandoned for an unknown reason. The success rate was 98% (49 successful procedures) in the midazolam group and 96% (48 successful procedures) in the melatonin group. Given the small number of failures, success differences between the two groups are unclear (Difference −0.02 (95% bootstrapped CI: −0.08 to 0.04)).

### Resource use and costs

Resource use costs are summarised in Table 3. IMP costs were higher for midazolam, as were costs of concomitant medications up to 14 days post procedure, with more children in the midazolam arm receiving either paracetamol (17 (34%) or ibuprofen (13 (26%) compared with those receiving melatonin (7 (14%) paracetamol; 5 (10%) ibuprofen). However, the confidence intervals between the groups overlapped. The incremental mean cost of midazolam is £46 more than melatonin over 14 days, however there is a large amount of uncertainty in the cost estimates. (Mean incremental cost -£46.20 95% bootstrapped CI -£66.74 to £166.14).

**Table 3:** Mean cost per group with 95% bootstrap confidence interval

	Melatonin	Midazolam	Incremental difference in costs and effects
N (%)	50	50	
Mean cost of IMP	£14.15	£68.93	
Mean cost of procedure	£327.20 (£296.20, £422.80)	£319.40 (£287.90, £419.90)	
Mean cost of concomitant medication during surgery	£116.10 (£76.50, £192.30)	£90.65 (£57.89, £174.34)	
Mean cost of concomitant medication up to 14 days	£24.04 (£9.19, £58.13)	£77.12 (£28.17, £220.76)	
Mean cost of other resources (GP appointment and out patient appointment)	0	£4.60 (£0.82, £15.12)	
Total cost (95% CI)	£474.60 (£455.30 to £646.20)	£520.80 (£414.10 to £577.90)	-£46.20 (-£166.14 to £66.74)
Number of successful procedures (%)	48 (96%)	49 (98%)	
Proportion successful (95% CI)	0.96 (0.90 to 1.00)	0.98 (0.94 to 1.00)	−0.02 (−0.08 to 0.04)

#### Primary analysis: cost-effectiveness analysis

**Fig. 1:**
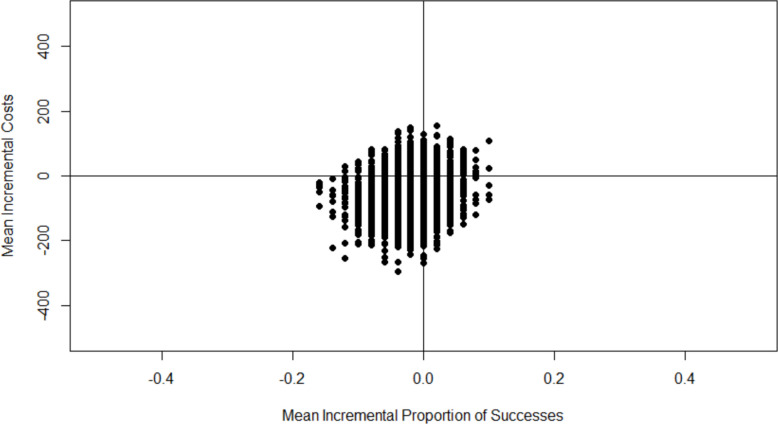
Comparison of melatonin with midazolam on the cost-effectiveness plane, cost per successful procedure

The bootstrap estimates of the costs and effects of melatonin compared with midazolam cover all four quadrants of the cost-effectiveness plane (Fig. 1), with most points (49%) being in the bottom left corner of the cost effectiveness plane showing melatonin is less costly but less effective than midazolam. Due to this uncertainty, with the bootstrap estimates covering all four quadrants of the cost effectiveness plane an incremental cost effectiveness ratio (ICER) is not calculated.

#### Secondary analysis: cost-utility analysis and cost-minimisation analysis

**Fig. 2:**
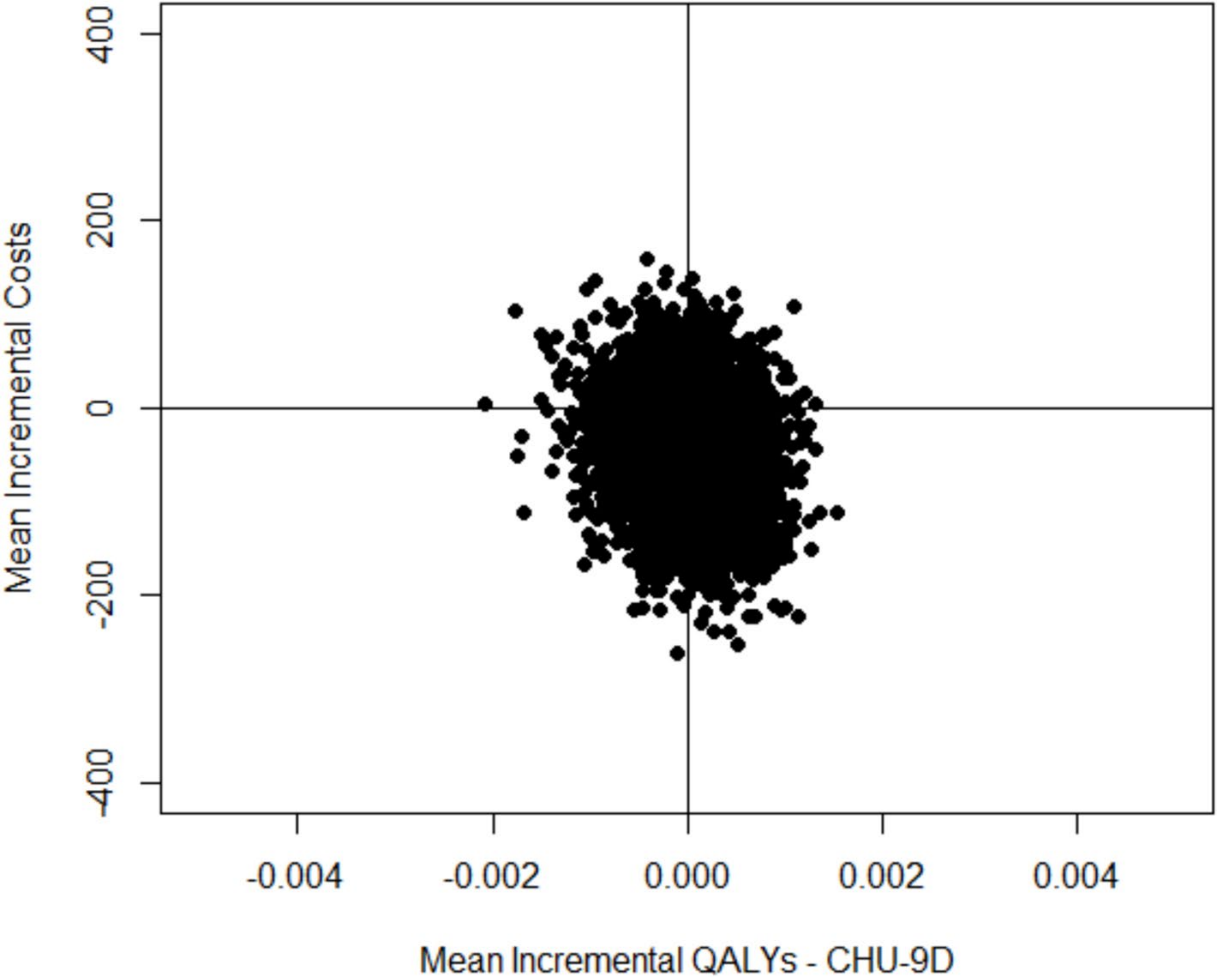
Comparison of melatonin with midazolam on the cost-effectiveness plane, cost per QALY

**Fig. 3:**
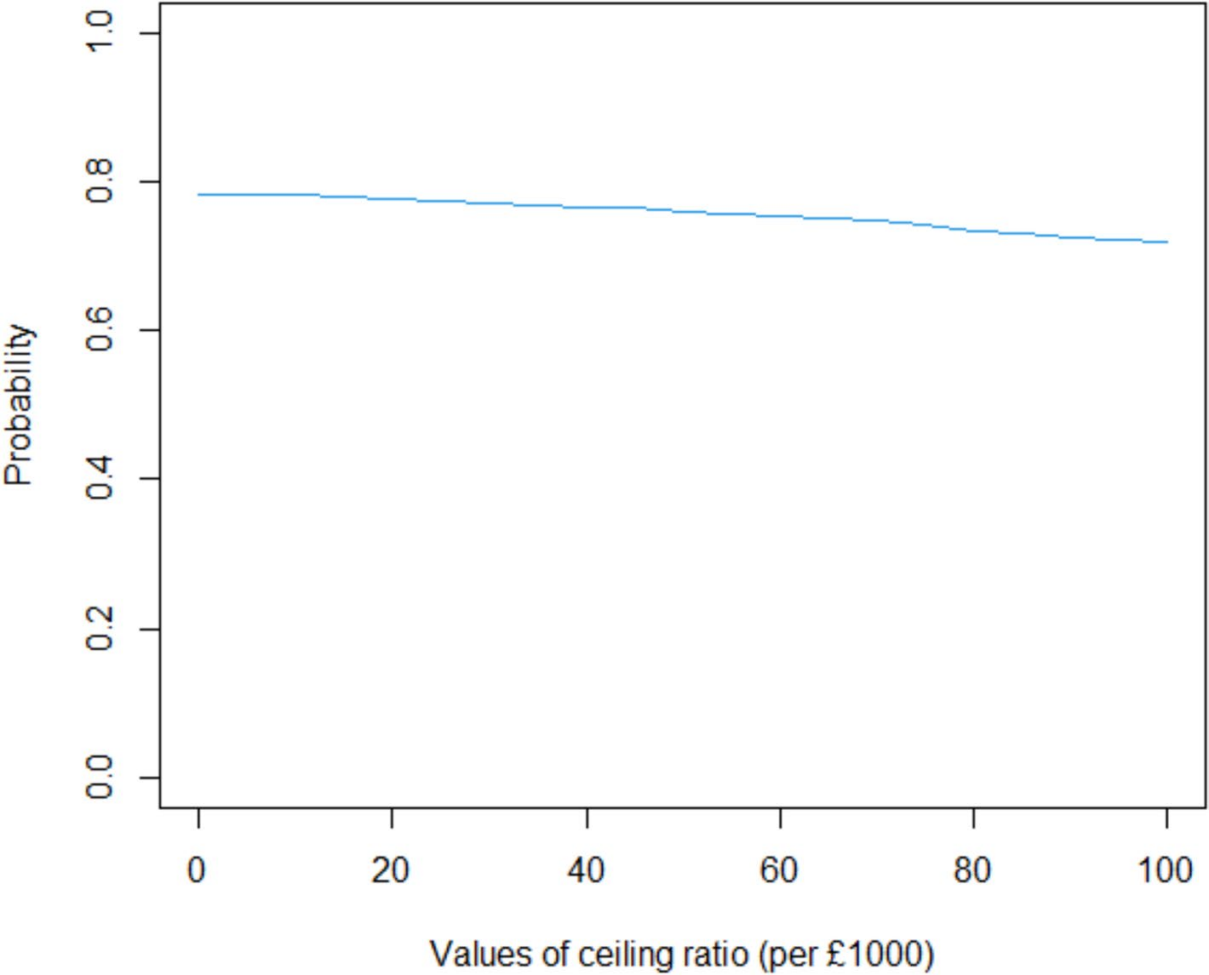
Cost-effectiveness acceptability curve for the comparison of melatonin with midazolam

Three secondary analyses are presented (Table 4). The first analysis presents the incremental cost per QALY for the whole sample and does not distinguish between proxy and self-reported QALY estimates for those under and over 7 years old. There is no difference in QALYs between the two IMP groups and results cover all 4 quadrants of the cost effectiveness plane with 46% of estimates being in the bottom right quadrant of the cost-effectiveness plane where melatonin is cheaper and more effective and 33% being in the bottom left quadrant of the cost-effectiveness plane where melatonin is cheaper and less effective (Fig. 2). Melatonin is 77.7% cost-effective at a willingness to pay threshold of £20,000 per QALY (Fig. 3).

**Table 4:** Secondary cost-effectiveness analysis: cost-utility analysis after imputing missing data overall and for those aged 7 and over and cost minimisation

	Melatonin	Midazolam	Incremental difference
Overall cost-utility analysis			
N	50	50	
QALY (95% CI)	0.0341 (0.330 to 0.0345)	0.0341 (0.0335 to 0.0346)	0 (−0.0008 to 0.0008)
Total cost (95% CI)	£474.60 (£455.30 to £646.20)	£520.80 (£414.10 to £577.90)	-£46.20 (-£166.14 to £66.74)
Cost-utility analysis age 7 and above			
N	30	36	
QALY (95% CI)	0.0339 (0.0319, 0.0345)	0.0339 (0.0332 to 0.0346)	0 (−0.001 to 0.001)
Total cost (95% CI)	£504.51 (£409.20 to £658.70)	£556.99 (£472.00 to £728.50)	-£52.48 (-£221.29 to £109.60)
Cost-minimisation analysis			
Total cost (95% CI)	£474.60 (£455.30 to £646.20)	£520.80 (£414.10 to £577.90)	-£46.20 (-£166.14 to £66.74)

The second analysis presents the incremental cost-effectiveness for those aged 7 and over, there is no difference in QALYs between the two IMP groups and results were uncertain where 43% of estimates showed melatonin to be cheaper and more effective and 31% of estimates showed melatonin is cheaper and less effective.

#### Cost minimisation

The third analysis was a cost-minimisation analysis (Table 4). This showed that, on average, midazolam was £46 more expensive compared with melatonin across a 14-day period, however this was not significant with a 95% bootstrapped confidence interval ranging from -£166 to £67.

### NHS and wider perspective

The main cost incurred by primary and secondary carers were due to loss of earnings (time away from employment or usual activities) over the study period (melatonin mean = £652 (95% CI: £391 to £1,128), midazolam mean = £638 (95% CI: £408 to £1,050)) (Table 6). Overall costs to secondary carers were similar for the two arms (melatonin mean £660 (95% CI: £398 to £1,135), midazolam mean £645 (95% CI: £416 to £1,060)) (Table 5).

**Table 5:** Summary of primary and secondary carer resource use and costs for IMP treatments

	Melatonin	Midazolam
N	50	50
Travel costs: Mean (95% CI)	£6.71 (£4.95 to £9.95)	£6.99 (£5.25 to £10.11)
Loss of earnings/time: Mean (95% CI)	£652 (£391 to £1,128)	£638 (£408 to £1,050)
Total travel and loss of earnings costs	£660 (£398 to £1,135)	£645 (£416 to £1,060)
Costs to NHS and primary and secondary carers	£1,134 (£846 to £1,606)	£1,166 (£918 to £1,588)

The mean cost to the NHS and primary and secondary carers over 14 days was similar for the two IMP arms (mean = £1,134 (95% CI: £846 to £1,606) melatonin: mean = £1,166 (95% CI: £918 to £1,588) midazolam). The mean incremental difference in costs between the two arms was -£32 (95% CI: -£502 to £450).

#### Subgroup analysis – cost-effectiveness analysis

As set out in the HEAP (SM1) cost-effectiveness analysis across subgroups was restricted to groups with a sample size of 30 or more, results are presented as incremental costs per successful procedure. For aged under 7 years there were less than 30 children per arm (*n* = 20 melatonin, *n* = 14 midazolam). For surgery specialty most children underwent head and neck procedures (dental, ENT or ophthalmology), with only 7 cases undergoing surgery for gastroenterology and MRI or other (*n* = 4 melatonin, *n* = 3 midazolam respectively). Therefore, cost-effectiveness analysis for subgroups is presented for those aged 7 or older and those undergoing head and neck surgery (Table 6).

**Table 6:** Summary of mean costs and proportion of successful procedures by age (≥ 7 years old) and those undergoing head and neck procedures (Dental, ENT and ophthalmology)

		Melatonin	Midazolam	Incremental difference in success rate
Age ≥ 7	N	30	36	
	Proportion successful	0.933	1.000	N/a
	Total cost (95% CI)	£504.51 (£413.30, £653.50)	£556.99 (£473.90, £732.80)	-£52.48 (-£221.29 to £109.60)
Head and neck procedures	N	46	47	
	Proportion successful (95% CI)	0.957 (0.891 to 1.00)	0.979 (0.936 to 1.00)	−0.022 (−0.09 to 0.04)
	Total cost (95% CI)	£485.80 (£420.70 to £596.20)	£531.00 (£460.30 to £660.50)	-£45.20 (-£170.39 to £76.37)

For those 7 years or older surgery was successful in 93% of those receiving melatonin and 100% of those receiving midazolam. It was not possible to look at the cost per successful procedure for those 7 years or over as all those receiving midazolam had a successful procedure.

The cost-effectiveness results for the head and neck surgery subgroup were similar to the overall results with most bootstrap cost-effectiveness estimates (60%) showing melatonin to be cheaper but less effective than midazolam but with a large amount of uncertainty.

## Discussion

This is the first study that has examined the cost-effectiveness of melatonin compared with midazolam in children, young people or adults. The results of this within trial study are inconclusive with no evidence suggesting that melatonin was cost-effective when compared with midazolam. However, the main findings of the trial suggested that melatonin was clinically inferior to midazolam [10].

Studies that have looked at the cost-effectiveness of midazolam have not been conclusive and may not be comparable with this study as they are not focused on its administration pre-medication. Wolf et al. [7] conducted an equivalence study comparing intravenous clonidine with intravenous midazolam in critically ill children in paediatric ICU, they found that clonidine was cheaper (£11,445 2011/12 prices £14,910 inflated to 2021/22 prices [27] than midazolam (£12,276 2011/12 prices £15,993 inflated to 2021/22 prices) though there was uncertainty in the estimates. Although this study examined costs over a 14-day period the results are not comparable as the children were in a different environment, being seen in paediatric ICU) compared to those in the MAGIC study (day case hospital visits). Yap et al.’s study [8] is also not comparable with MAGIC as it examined the cost-effectiveness of a combination of midazolam and droperidol with droperidol or olanzapine in adults with acute agitation in the emergency department. Further, Hohl et al. [6] compared propofol with midazolam in adults in the emergency department. In 2010 the National Clinical Guideline Centre produced a set of guidelines for sedation in children and young people; in developing the guidelines the authors constructed a number of cost-effectiveness models for alternative procedures. For each procedure the alternative sedation strategies were compared with general anaesthetic and in all cases, it was concluded that sedation was cost-effective [28].

Although the low number of abandoned procedures in either arm (2 in the melatonin arm and 1 in the midazolam arm) indicates that both drugs had anxiolytic effects [10]. Based on the results of this trial there are no or extremely limited indications for the use of melatonin rather than midazolam as a premedication in children or young people. The absence of adverse events attributable to midazolam and its safe routine use reinforces this view.

A qualitative study conducted as part of the Magic trial suggested research such as a discrete choice experiment could explore the attributes of premedication important to children, caregivers and clinicians to ensure that any proposed experimental treatments are acceptable within this population and to prescribing clinicians [29]. This study could also identify where a new proposed premedication may best fit within the population i.e. subgroups here it may be best (or worst) placed.

### Strengths and weaknesses of the analysis

This is the first study to examine the cost-effectiveness of melatonin with midazolam in children and young people pre-medication before a general anaesthetic. As the study closed early it is limited by the smaller sample size and the low number of unsuccessful procedures limiting the certainty of the results.

Further, a typical, economic evaluations present cost-utility analysis as the primary analysis. However, QALYs were not selected as the primary analysis due to the short (14 day) time frame of the study and the lack of evidence that QALYs would change over this timeframe and due to not having a validated measure of QALYs in children across the age range of the study. Given the aim of the medications is a reduction in anxiety before general anaesthetic is short-term QALYs may not be an appropriate measure as they are not sensitive enough to show a change in health-related quality of life if one exists. Our study showed that the QALY difference was very small, suggesting this may not be a sensitive measure for pre-medication studies.

A further consideration in evaluating cost-effectiveness in paediatric populations is the selection of a health-related quality of life measure that can be used to obtain QALYs across the age range of the study. THE CHU-9D has been validated for use in children aged 7 to 17 with a proxy version for children under 7 [17, 30]. In MAGIC the proxies completed the CHU-9D for children under 7 and the children aged 7 or older self-completed the questionnaire. Proxy completers tend to underestimate the health-related quality of life of the person they are completing for [18] and there is evidence suggesting further work is needed on the validity of the CHU-9D in children 5 or younger [31]. Therefore, there is uncertainty in using the CHU-9D across the age range observed in the MAGIC study.

Both cost-effectiveness (cost per successful procedure) and cost-utility analysis showed uncertainty in the cost-effectiveness results. In addition, there was a large amount of missing QALY data (43%) likely adding to the uncertainty in the results. It is possible that, had the study recruited to target sample size and not stopped early that more definitive results would have been observed. However, the incremental difference in the proportion of successes and QALYs was small suggesting no effect so uncertainty may have remained. Cost minimisation analysis over 14 days showed melatonin to be, on average, slightly less expensive than midazolam, though results were uncertain.

As outlined, in the methods, the cost-effectiveness analysis presented here deviated from the health economic analysis plan (HEAP) to use a decision analytic model to explore the cost-effectiveness of melatonin over a 1-year time frame (SM1). However, the main trail results [10] and the within-trial cost-effectiveness analysis did not support melatonin being cost-effective resulting in a decision not to carry out the modelling.

## Conclusions

In children with preoperative anxiety, midazolam is more clinically effective premedication than melatonin [10]. The results of our study were inconclusive showing no evidence that melatonin was more cost-effective than midazolam. The study closed early owing to issues with recruitment and this has limited the economic analysis as a smaller sample size restricted the subgroup analysis and the high number of successful procedures meant that a longer-term cost-effectiveness model was not possible and unlikely to demonstrate cost-effective results.

## Supplementary Information

Supplementary Material 1.

Supplementary Material 2.

Supplementary Material 3.

## Data Availability

All data requests should be submitted to the corresponding author for consideration. Access to anonymised data may be granted following review.

## Abbreviations

CHU-9D: Child health utility 9D
ENT: Ear, nose and throat
FMI: Fraction of missing information
GBP: Great British pounds
HRQoL: Health related quality of life
HEAP: Health economic analysis plan
ICER: Incremental cost-effectiveness ratio
IMP: Investigational medicinal product
MAGIC: Melatonin for anxiety prior to general anaesthesia in children
mYPAS-SF: Modified Yale preoperative anxiety scale – short form
PAED: Paediatric anaesthesia emergence delirium
PSS: Personal and social services
QALY: Quality adjusted life year
RCT: Randomised controlled trial
SM: Supplementary material
