# Supplementary material for: A cost-effectiveness analysis of melatonin in comparison with midazolam for anxiety prior to general anaesthesia in children: the MAGIC randomised controlled trial (melatonin for anxiety prior to general anaesthesia in children)
Source: BMC Anesthesiol. 2025 Dec 17;26:47. doi: 10.1186/s12871-025-03489-x (PMC12821929; doi:10.1186/s12871-025-03489-x)
Supplement: Supplementary file 1 — Supplementary Material 1. [file 12871_2025_3489_MOESM1_ESM.pdf]

The MAGIC trial (Melatonin for Anxiety prior to General anaesthesia In Children)

HEALTH ECONOMICS ANALYSIS PLAN

VERSION 2.0 (02-May-2023)

1 Contents

2 Administrative Information 4

1.1 HEAP Administrative Information 4

3 Abbreviations 5

4 Trial Introduction & Background 6

4.1 Trial Background and Rationale 6

4.2 Aim of the Trial 6

4.3 Objectives of the trial 6

4.3.1 Feasibility objectives: 6

4.3.2 Clinical objectives 6

4.3.3 Economic objectives 7

4.4 Trial design 7

4.5 Trial population 7

4.6 Intervention and comparators 8

4.7 Trial start and end dates 8

5 Economic Approach 8

5.1 Aims of economic evaluation 8

5.2 Objectives of economic evaluation 8

5.3 Overview of economic analysis 9

5.4 Jurisdiction 9

5.5 Perspectives 9

5.6 Time horizon 9

6 Economic Data Collection and Management 9

6.1 Statistical software use for health economic analysis 9

6.2 Identification of resources 10

6.3 Measurement of resource use data 10

6.4 Valuation of resource use data 10

6.5 Identification of outcomes 11

6.6 Measurement of outcomes 11

6.7 Valuations of outcomes 11

7 Economic Data Analysis 12

7.1 Analysis population 12

7.2 Timing of analyses 12

7.3 Discount rates for costs and benefits 12

7.4 Cost-effectiveness threshold(s) 12
7.5 Statistical decision rule(s) 12
7.6 Analysis of costs 12
7.7 Analysis of outcomes 12
7.8 Data cleaning for analysis 12
7.9 Missing data 13
7.10 Analysis of cost-effectiveness 13
7.11 Sampling uncertainty 13
7.12 Subgroup analyses 13
7.13 Sensitivity Analyses 14
8 Modelling and VOI Analyses 14
8.1 Decision analytic modelling 14
8.2 Model type 14
8.3 Model structure 14
8.4 Treatment effect beyond the end of the trial 16
8.5 Other key assumptions 16
8.6 Methods for identifying and estimating parameters 16
8.7 Model uncertainty 16
8.8 Model validation 16
8.9 Subgroup analyses/ Heterogeneity 16
9 Reporting/Publishing 16
9.1 Reporting standards 16
9.2 Reporting deviations from the HEAP 17
10 References 18

2 Administrative Information

1.1 HEAP Administrative Information

Title	The MAGIC trial (Melatonin for Anxiety prior to General anaesthesia In Children): A Multicentre, Parallel Randomised Controlled Trial of Melatonin Versus Midazolam in the Premedication of Anxious Children Attending for Elective Dental, Ophthalmologic or ENT Surgery Under General Anaesthesia
Trial registration number; registry	ISRCTN 18296119
Source of funding	NIHR HTA (project number 16/80/08)
Purpose of HEAP	The purpose of this HEAP is to describe the analysis and reporting procedure intended for the economic analyses to be undertaken. The analysis plan is designed to ensure that there is no conflict with the protocol and associated statistical analysis plan and it should be read in conjunction with them
Trial protocol version; date	This document has been written based on information contained in the trial protocol version 4.1 dated 28/Sep/2020
Trial Statistical Analysis Plan (SAP) version, date	This document has been written based on information contained in the trial statistical analysis plan version 3, 29/Nov/2022
Trial HEAP version, date	2, 02/May/2023
HEAP revisions	Version 1 updated to version 2 to reflect updated eligibility criteria.
Roles and responsibilities	This HEAP was prepared by Dr Laura Flight (junior health economist) and Professor Tracey Young (senior health economist) and approved by Prof Simon Dixon (Independent Senior Health Economist). The trial health economist(s) are responsible for conducting and reporting the economic evaluation in accordance with the HEAP.

APPROVALS ***The following people have reviewed the Health Economics Analysis Plan and are in agreement with the contents.***

**Role**	**Name**	**Signature**	**Date**
Author	Dr Laura Flight & Prof. Tracey Young		02/May/2023
Independent Health Economist	Prof Simon Dixon		03/May/2023
Chief Investigator	Prof Chris Deery		05/May/2023

3 Abbreviations

ASA	American Society of Anaesthesiologists
CEA	Cost-effectiveness Analysis
CEAC	Cost-effectiveness Analysis Curve
CHU-9D	Child Health Utility 9D
ENT	Ear, Nose and Throat
FPS-R	Faces Pain Scale – Revised
GBP	Great British Pounds
HRQoL	Health Related Quality of Life
ICER	Incremental Cost-effectiveness Ratio
ITT	Intention to Treat
mYPAS-SF	Modified Yale Preoperative Anxiety Scale – Short Form
PAED	Paediatric Anaesthesia Emergence Delirium
PHBQ-AS	Post Hospitalization Behaviour Questionnaire for Ambulatory Surgery
PSS	Personal and Social Services
QALY	Quality Adjusted Life Year
RCT	Randomised Controlled Trial
VSRS	Vancouver Sedation Recovery Scale

4 Trial Introduction & Background

4.1 Trial Background and Rationale

The hospital anaesthetic room is a worrying place for a child, and reducing their distress leads to a better overall experience, and improves recovery from the anaesthetic, reduces pain after surgery and avoids unnecessary reappointments and delays to operations. Currently, those children with high levels of distress are recommended a "premedication"; that is, a medicine to reduce anxiety ahead of surgery. Midazolam – the current premedication for an anxious child needing an anaesthetic – is effective, although it has many side effects including loss of coordination and risks to breathing. Midazolam can also have unpredictable effects on anxiety, with some children becoming overexcited rather than being calmed. Melatonin, which also has anxiolytic properties, offers an alternative calming medicine, has shown promise as it avoids midazolam's side effects.

4.2 Aim of the Trial

The main aim of this study is to evaluate the clinical non-inferiority and cost-effectiveness of melatonin, and to assess melatonin's side-effects profile compared to midazolam in the premedication of anxious children prior to general anaesthesia for elective ENT, ophthalmological, dental, gastroenterology, radiology, plastic, orthopaedic, urology and other general surgery.

4.3 Objectives of the trial

4.3.1 Feasibility objectives:

To undertake an internal pilot trial to determine the feasibility of a full-scale trial, in terms of:

- Recruitment
- Retention
- Allocation concealment and blinding

4.3.2 Clinical objectives

4.3.2.1 Efficacy

To evaluate if melatonin, in relation to midazolam is:

- Non-inferior in dealing with pre-operative anxiety evaluated by mYPAS-SF score over the following three standard preoperative time points recommended for the scale:
    - Start of transfer to theatre.
    - Entry to anaesthetic room.
    - Administration of anaesthesia.

- Superior in dealing with secondary efficacy outcomes (anaesthetic turnaround time, recovery time).
- Non-inferior in dealing with the secondary efficacy outcome (anaesthetic failure rate).

4.3.2.2 *Harms and Safety*

- To evaluate if melatonin, in relation to midazolam is superior in dealing with secondary safety. outcomes (PAED, VSRS, FPS-R, analgesia requirements, PHBQ-AS, adverse events, orientation and cognitive/psychomotor function).
- To describe Serious Adverse Events data (summarised both at patient level and event level) and report listings between the different arms.

4.3.3 Economic objectives

To estimate the cost-effectiveness of introducing melatonin compared to usual care (midazolam), over the study period and modelled to one year using both a cost per successful procedure and cost-per QALY approach.

4.4 Trial design

MAGIC is a parallel group, double blind, randomised controlled trial (RCT) to evaluate the non-inferiority of melatonin against midazolam in dealing with pre-operative anxiety in children undergoing surgery. The study will be conducted in twenty large NHS trusts and participants will be randomised to receive either midazolam or melatonin in the ratio 1:1. The trial has been designed with an internal pilot phase during the first 6 months of active recruitment, which will assess the feasibility aspects of conducting the main trial as guided by pre-planned STOP/GO.

4.5 Trial population

Inclusion Criteria:

1. Children aged 3-14 years
2. Children undergoing elective dental, ophthalmological, ENT, gastroenterology, radiology, plastic, orthopaedic, urology or other general surgery under general anaesthesia.
3. Pragmatically assessed by healthcare professionals as requiring premedication as per local standard care for high/expected high levels of preoperative distress prior to surgery.
4. American Society of Anaesthesiologists (ASA) grades I & II.
5. Parent or person with parental responsibility able to give written, informed consent.

Exclusion Criteria:

1. Not undergoing elective, day-case surgery under general anaesthesia.

2. Not displaying level of anxiety that would usually warrant premedication under the standard NHS care pathway.
3. Reason for premedication other than anxiety.
4. Current prescription of melatonin, midazolam or other non-permitted drug (see Section 7.11.2 of the protocol).
5. Obstructive sleep apnoea.
6. ASA grades III, IV & V.
7. Severe learning disability rendering child unable to communicate even with specialised support.
8. Child verbally declines to participate in the trial.

4.6 Intervention and comparators

Intervention: 0.5 mg/kg melatonin (max 20 mg) 30 mins prior to transfer to theatre

Comparator: 0.5 mg/kg midazolam (max 20 mg) 30 mins prior transfer to theatre

4.7 Trial start and end dates

The trial started recruitment in July 2019, and was suspended in March 2020 due to the Covid-19 pandemic. The trial re-opened to recruitment in October 2020 and June 2022.

5 Economic Approach

5.1 Aims of economic evaluation

The health economic evaluation aims to assess the cost-effectiveness of introducing melatonin, compared to usual care (midazolam), over the study period and modelled to one year using both a cost per successful procedure and cost-per quality adjusted life year (QALY) approach.

5.2 Objectives of economic evaluation

The primary objective of the health economic evaluation is a cost-effectiveness analysis using the resource use and the number of successful procedures undertaken over the study period; comparing immediate release oral melatonin with standard care (oral midazolam). A decision tree model will be developed to estimate the cost-effectiveness over a one-year period.

Given that QALYs will be collected over a short time period and it is unclear whether sedation has long-term effects on quality of life, this analysis will not be used as a primary analysis but the cost per QALY will be examined in secondary analysis (1). Therefore, a secondary objective is a cost - utility

analysis that looks at costs per quality adjusted life year using the CHU-9D questionnaires reported (2).

5.3 Overview of economic analysis

The within trial analysis will be performed using individual level data from the MAGIC trial. The analytical approaches will take the form of cost-effectiveness and cost-utility analyses. Based on trial evidence, incremental cost-effectiveness (and cost-utility) ratios will be calculated by taking a ratio of the difference in the mean costs and mean effects (or utility measure).

A decision tree will be constructed to explore the cost-effectiveness of melatonin over a one-year period. This model will follow a similar structure to that by the National Clinical Guideline Centre that looked at sedation in children and young people for diagnostic therapies (1). As with the trial-based analysis, results will be presented in terms of an ICER.

5.4 Jurisdiction

The trial will be conducted in the UK setting, which has a national health service (NHS), providing publicly funded healthcare, primarily free of charge at the point of use.

5.5 Perspectives

The primary analysis will take a NHS and Personal Social Services (PSS) perspective. A secondary analysis will also consider a wider perspective that will include costs incurred by parents/carers.

5.6 Time horizon

The primary economic analysis will compare the costs and number of successful procedures over the study period (14 days). A decision tree will then be used to estimate the cost-effectiveness over the one-year period following randomisation.

6 Economic Data Collection and Management

6.1 Statistical software use for health economic analysis

R version 2022.07.0 or higher (3) and Stata version 17 or higher (4) will be used for exploratory analyses and for the main statistical analysis.

6.2 Identification of resources

The costing approach will include identification of resource use, measurement and valuation(5). Resource use data will be collected as part of the trial at baseline and 14 day follow-up using a specially developed case report form, completed by the Research Nurse on behalf of the caregiver, completion will be either face-to-face or over the phone.

Health care resource use that may differ between arms will be measured including, but not limited to: health service resource use and primary and secondary caregiver personal costs.

6.3 Measurement of resource use data

Relevant health care resource use captured during the trial will include clinical time, anaesthetic time, recovery time and medication costs including premedication costs and also pain and anti-emetic medication used as in-patient and at discharge. To capture all relevant aspects of resource use in the trial the following case report forms are used:

- Baseline Participants (Child Health Utility 9D)
- General Surgery details (clinical, anaesthetic, recovery time and concomitant medications)
- 14 day follow-up (Child Health Utility 9D)
- Adverse event log
- Concomitant medications pre and post admission
- Caregiver costs (baseline, 14 days – time off work, travel costs)
- Equipment used to deliver medications

6.4 Valuation of resource use data

Each of the resource use items will be valued in monetary terms, where costs are not provided by the participants appropriate unit costs from standard health economic sources such as

- British National Formulary (6)
- NHS Reference costs (7)
- PSSRU Unit costs of health and social care (8)
- NHS Agenda for Change (2022)
- Office of National Statistics annual survey of hours and earnings (2022).

Costs are calculated using Great British Pounds (GBP) for the year of analysis, where costs were not available for this year they were inflated using the hospital and community health services (HCHS) index (8)

6.5 Identification of outcomes

The primary economic outcome measure will be number of successful procedures over the study duration. The anaesthetic failure rate for this trial is defined to be the proportion of patients for whom surgery is abandoned before the point of unconsciousness. A successful procedure is defined by surgery not being abandoned before the point of unconsciousness.

The secondary economic outcome measure will be Quality Adjusted Life Years (QALYs). This combines both the quality and quantity of life and is measured using utility (9). Utility quantifies health related quality of life (HRQoL) and is a value anchored between zero and one, where one represents full health and zero represents death (9). Utilities below zero represent states worse than death. Utilities in the MAGIC trial will be derived from utility scores, obtained using the Child Health Utility 9D (CHU-9D) questionnaire (2). Measured domains include worried, sad, pain, tired, annoyed, schoolwork/homework, sleep, daily routine and activities.

6.6 Measurement of outcomes

The research nurse will record the success of a procedure on the General Surgery Details CRF. The time for point of consciousness and the time of abandonment during transfer and surgery will also be recorded.

The CHU-9D will be used to measure quality of life at baseline and 14 days. This is completed by the parent for children aged 5-6 and by the child for children aged 7-14 (2).

6.7 Valuations of outcomes

A utility score is calculated by assigning utility values to each response and then summing these values. A CHU-9D score can only be calculated if all questions have been answered. The CHU-9D utility score ranges from 0.33 to one where higher scores represent greater health related quality of life (2). QALYs will be estimated from utility scores at baseline and subsequent follow-up time points using linear interpolation.

The CHU-9D has not been shown to be valid in the 3-4 age group. This will be noted as a potential limitation in the discussion of the health economic analysis. An additional question is included on the questionnaire for the under 5s *How would you rate your child's health today?* Responses to this question will be used to validate the overall CHU-9D score in this age group if required.

7 Economic Data Analysis

7.1 Analysis population

The full analysis set will include all randomised participants, which is in accordance with the "intention to treat" (ITT) principle.

7.2 Timing of analyses

The primary analysis will be conducted once all patients have been followed for 14 days after randomisation. The final analysis will include the within-trial analysis and model-based analysis taking a one-year time horizon.

7.3 Discount rates for costs and benefits

No discounting will be applied as only 12 month data will be used in the analysis.

7.4 Cost-effectiveness threshold(s)

A range of willingness to pay thresholds will be considered for the cost-utility analyses, including the NICE threshold of £20,000 per QALY, as per NICE guidance (10).

7.5 Statistical decision rule(s)

Mean differences in costs, QALYs, number of successful procedures, net benefits and incremental cost-effectiveness ratio (ICER) will be estimated with associated 95% confidence intervals.

7.6 Analysis of costs

Histograms of total costs for complete case data will be used to visualise the distribution of cost data. Mean total costs in each arm will be summarised and then broken down into the different components of resource use to identify the drivers in the total costs.

7.7 Analysis of outcomes

The mean number of successful procedures in each arm, over the study period, will be calculated.

Mean QALY at each time point will be plotted visually using a line plot to illustrate the pattern of utility over the trial period for each intervention. Mean QALY will be calculated using linear interpolation between time points. This will be stratified by participant age (less than seven years and greater than or equal to seven years) to reflect the different modes of completion.

7.8 Data cleaning for analysis

Data will be checked for face validity and any unusual results queried with the study team. Any changes to the data will be documented and implemented using R code. No changes will be made to the original data.

7.9 Missing data

Missing data can give misleading estimates of a within-trial cost-effectiveness analysis. A complete-case analysis uses only participants with no missing data in the key cost and benefit outcomes. This is undesirable as it reduces the sample size and affects the power of the study (11). If required, patterns of missing data will be assessed using the approach outlined by Faria et al (2014) (11)and will include a descriptive analyses of:

1. Proportion of missing data by treatment arm, at each follow-up period, to assess whether or not missing data differed by arm.
2. Missing data patterns to determine whether or not data were missing for all items or individual items of utility scores and resource use items over the trial follow-up.

If deemed to be appropriate multiple imputation will be used to impute missing values. The number of imputations will be based on the highest percentage of missing data for the variables of interest. The imputation will be performed per randomisation arm, for all imputed variables, except baseline covariates with missing data, for which imputation will be performed across all observations.

7.10 Analysis of cost-effectiveness

Cost-effectiveness analysis (CEA) will be performed to compare the cost-effectiveness of melatonin to midazolam. A regression model will be used to estimate the difference in mean total costs and number of successful procedures between treatment arms, for all patients regardless of age or procedure. The costs and number of successful procedures will be combined to calculate an incremental cost-effectiveness ratio (ICER).

7.11 Sampling uncertainty

To illustrate uncertainty cost-effectiveness confidence ellipses will be produced. The willingness to pay threshold per unit of effectiveness will be varied to assess the uncertainty associated with the estimates. Additionally, a cost-effectiveness acceptability curve (CEAC) will be constructed illustrating the probability of each treatment being most cost-effective for a range of threshold values.

7.12 Subgroup analyses

Analyses will be conducted on the final dataset to investigate how cost-effectiveness varies between different patient subgroups including: surgical specialty (head and neck, gastro and MRI, other) and age (<7 years, ≥7 years). Any subgroup analyses for which the smaller subgroup includes fewer than 30 participants will be omitted.

7.13 Sensitivity Analyses

A number of sensitivity analyses will be performed to assess the robustness of the within-trial health economic estimates. In each case a similar regression model will be used to estimate differences in total costs, differences in effects and the ICER. Subgroup analyses will be reported if appropriate. The analyses include:

- A complete case analysis will be carried out to include all randomised participants that have complete data on cost and effectiveness outcomes.
- Cost-utility analysis
- Incorporating caregiver costs

8 Modelling and VOI Analyses

8.1 Decision analytic modelling

Decision analytic modelling will be undertaken to explore the cost-effectiveness of melatonin over 1-year after randomisation, irrespective of the statistical significance of the trial results, if there is the potential for the cost-effectiveness of melatonin to improve cost-effectiveness under a longer analysis time horizon than the 14 day follow-up. A decision tree model will be used to extrapolate the costs and outcomes beyond the 14-day follow-up period of the study.

8.2 Model type

A decision tree will be constructed to explore the cost-effectiveness of melatonin over a 1-year period.

8.3 Model structure

A search strategy adopted from the National Clinical Guideline Centre that looked at sedation in children and young people for diagnostic therapies [1], will be used to identify recent, cost-effectiveness studies.

The health economic model will follow a similar structure to the models proposed by the National Clinical Guideline Centre and any identified models (Figure 1). This structure will then be discussed with clinical experts to capture relevant events in the 12 months following sedation.

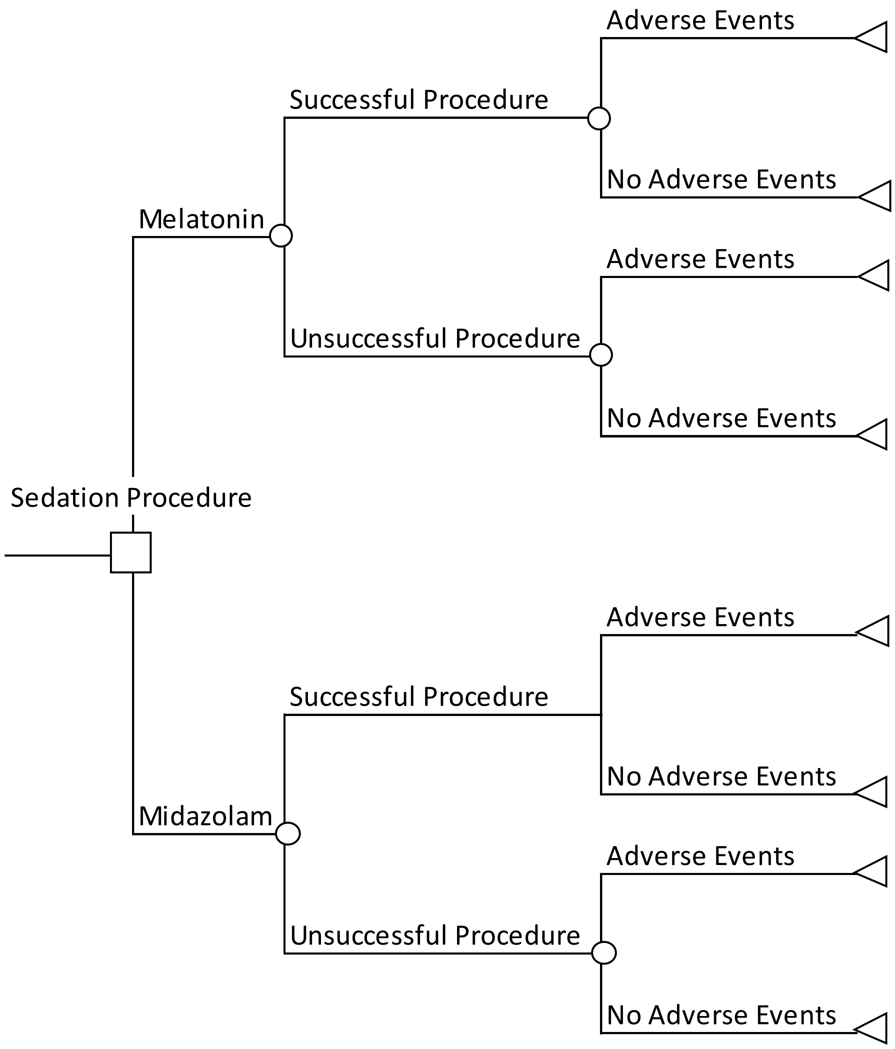

-

Figure 1 Example of potential decision tree for the MAGIC health economic analysis

8.4 Treatment effect beyond the end of the trial

The number of successful procedures from the trial will be used to estimate the probability of success or failure in each treatment arms.

8.5 Other key assumptions

No discounting will be applied as the model is only considering a 1 year time frame. The model structure may be subject to change following initial exploratory analysis of the trial data.

8.6 Methods for identifying and estimating parameters

The model will require three main sets of parameters, which are expected to be mainly derived from the trial data. 1) The number of successful procedures from the trial will be used to estimate the probability of success of failure in each treatment arms. 2) The same probability will be used for any repeat procedures following the first failure. 3) Cost of each procedure will be estimated from trial data (resource use).

8.7 Model uncertainty

Parameter uncertainty will be assessed using probabilistic sensitivity analysis (i.e. by fitting a probability distribution to each uncertain parameter) and running Monte Carlo simulations

8.8 Model validation

The model will be estimated and internally validated using trial data, it will be compared with other published studies and discussed with the study team.

8.9 Subgroup analyses/ Heterogeneity

The model will be used to evaluate the cost effectiveness of melatonin in comparison with midazolam across surgical specialty (head and neck, gastro and MRI, other) and age (<7 years, ≥7 years).

9 Reporting/Publishing

9.1 Reporting standards

The results of the within trial analysis will be reported in line with the CHEERS checklist for reporting economic evaluations (12).

This HEAP was written using the template provided Thorn et al (2020) (13).

9.2 Reporting deviations from the HEAP

Any deviation from HEAP will be described and justified in the final published report.

10 References

1. National Clinical Guideline Centre UK. Sedation in Children and Young People: Sedation for Diagnostic and Therapeutic Procedures in Children and Young People. 2010.

2. Stevens K. Valuation of the Child Health Utility 9D Index. Pharmacoeconomics. 2012;30(8):729–47.

3. RStudio Team (2022). RStudio: Integrated Development for R. RStudio, PBC, Boston, MA URL. Available from: http://www.rstudio.com/

4. StataCorp. Stata Statistical Software: Release 17. College Station, TX: StataCorp LLC; 2021.

5. Drummond M, Brandt A, Luce B, Rovira J. Standardizing methodologies for economic evaluation in health care. Int J Technol Assess Heal Care. 1993;9(1):26–36.

6. Joint Formulary Committee. British National Formulary (online) [Internet]. Available from: http://www.medicinescomplete.com

7. Department of Health. Reference Costs 2020/21. [Internet]. 2023. Available from: https://www.england.nhs.uk/costing-in-the-nhs/national-cost-collection/

8. Jones KC, Weatherly H, Birch S, et al. Unit Costs of Health and Social Care Manual 2022. Project report [Internet]. 2023. Available from: https://kar.kent.ac.uk/100519/

9. Drummond M, Sculpher M, Claxton K, Stoddart G, Torrance G. Methods for the economic evaluation of health care programmes. Oxford University Press; 2015.

10. National Institute for Health and Care Excellence. NICE health technology evaluations: the manual [Internet]. 2023. Available from: https://www.nice.org.uk/process/pmg36/chapter/introduction-to-health-technology-evaluation

11. Faria R, Gomes M, Epstein D, White IR. A guide to handling missing data in cost-effectiveness analysis conducted within randomised controlled trials. Pharmacoeconomics. 2014;32(12):1157–70.

12. Husereau D, Drummond M, Petrou S, Carswell C, Moher D, Greenberg D. Consolidated health economic evaluation reporting standards (CHEERS) statement. Cost Eff Resour Alloc. 2013;11(1):6.

13. Thorn J, Davies C, Brookes S, Noble S, Dritsaki M, Gray E, et al. Content of Health Economics Analysis Plans (HEAPs) for Trial-Based Economic Evaluations: Expert Delphi Consensus Survey. Value Heal. 2020;
