# Supplementary material for: A cost-effectiveness analysis of melatonin in comparison with midazolam for anxiety prior to general anaesthesia in children: the MAGIC randomised controlled trial (melatonin for anxiety prior to general anaesthesia in children)
Source: BMC Anesthesiol. 2025 Dec 17;26:47. doi: 10.1186/s12871-025-03489-x (PMC12821929; doi:10.1186/s12871-025-03489-x)
Supplement: Supplementary file 3 — Supplementary Material 3. [file 12871_2025_3489_MOESM3_ESM.docx]

CONSORT Checklists for The MAGIC trial: a pragmatic , parallel, noninferiority , randomised trial of melatonin versus midazolam in the premedication of anxious children attending for elective surgery under general anaesthetic. British Journal of Anaesthesia 132(1): 76-85 <https://pmc.ncbi.nlm.nih.gov/articles/PMC10797512/pdf/main.pdf>

Checklists for Pragmatic trials and non-inferiority trials with page numbers and sections are set out below.

Prepared by Tracey Young 23/06/2025

Extension to CONSORT Checklist for pragmatic trials

<https://pubmed.ncbi.nlm.nih.gov/19001484/>

| **Section** | **Item** | **Standard CONSORT description** | **Extension for pragmatic trials** | **Reported in Section** |
| --- | --- | --- | --- | --- |
| Title and abstract | 1 | How participants were allocated to interventions (eg, “random allocation,” “randomised,” or “randomly assigned”) |  | Title and abstract |
| **Introduction** |  |  |  |  |
| Background | 2 | Scientific background and explanation of rationale | Describe the health or health service problem that the intervention is intended to address and other interventions that may commonly be aimed at this problem | Background |
| **Methods** |  |  |  |  |
| Participants | 3 | Eligibility criteria for participants; settings and locations where the data were collected | Eligibility criteria should be explicitly framed to show the degree to which they include typical participants and/or, where applicable, typical providers (eg, nurses), institutions (eg, hospitals), communities (or localities eg, towns) and settings of care (eg, different healthcare financing systems) | Methods: Study design and participants  Methods: Randomisation and blinding  Methods: Data collection  Methods: Interventions |
| Interventions | 4 | Precise details of the interventions intended for each group and how and when they were actually administered | Describe extra resources added to (or resources removed from) usual settings in order to implement intervention. Indicate if efforts were made to standardise the intervention or if the intervention and its delivery were allowed to vary between participants, practitioners, or study sites | Methods: Study design and participants  Methods: Randomisation and blinding  Methods: Data collection  Methods: Interventions |
|  |  |  | Describe the comparator in similar detail to the intervention | Methods: Interventions |
| Objectives | 5 | Specific objectives and hypotheses |  |  |
| Outcomes | 6 | Clearly defined primary and secondary outcome measures and, when applicable, any methods used to enhance the quality of measurements (eg, multiple observations, training of assessors) | Explain why the chosen outcomes and, when relevant, the length of follow-up are considered important to those who will use the results of the trial | Methods:  Primary outcome measure  Secondary outcome measures |
| Sample size | 7 | How sample size was determined; explanation of any interim analyses and stopping rules when applicable | If calculated using the smallest difference considered important by the target decision maker audience (the minimally important difference) then report where this difference was obtained | Methods: Sample size |
| Randomisation—sequence generation | 8 | Method used to generate the random allocation sequence, including details of any restriction (eg, blocking, stratification) |  | Methods: Randomisation and blinding |
| Randomisation—allocation concealment | 9 | Method used to implement the random allocation sequence (eg, numbered containers or central telephone), clarifying whether the sequence was concealed until interventions were assigned |  | Methods: Randomisation and blinding |
| Randomisation—implementation | 10 | Who generated the allocation sequence, who enrolled participants, and who assigned participants to their groups |  | Methods: Randomisation and blinding |
| Blinding (masking) | 11 | Whether participants, those administering the interventions, and those assessing the outcomes were blinded to group assignment | If blinding was not done, or was not possible, explain why | Not applicable |
| Statistical methods | 12 | Statistical methods used to compare groups for primary outcomes; methods for additional analyses, such as subgroup analyses and adjusted analyses |  | Methods: Statistical analysis |
| **Results** |  |  |  |  |
| Participant flow | 13 | Flow of participants through each stage (a diagram is strongly recommended)—specifically, for each group, report the numbers of participants randomly assigned, receiving intended treatment, completing the study protocol, and analysed for the primary outcome; describe deviations from planned study protocol, together with reasons | The number of participants or units approached to take part in the trial, the number which were eligible, and reasons for non-participation should be reported | Results: Figure 1 |
| Recruitment | 14 | Dates defining the periods of recruitment and follow-up |  | Results |
| Baseline data | 15 | Baseline demographic and clinical characteristics of each group |  | Results & Table 1 |
| Numbers analysed | 16 | Number of participants (denominator) in each group included in each analysis and whether analysis was by “intention-to-treat”; state the results in absolute numbers when feasible (eg, 10/20, not 50%) |  | Results, Figures 1-4, Table 1 & 2 |
| Outcomes and estimation | 17 | For each primary and secondary outcome, a summary of results for each group and the estimated effect size and its precision (eg, 95% CI) |  | Results |
| Ancillary analyses | 18 | Address multiplicity by reporting any other analyses performed, including subgroup analyses and adjusted analyses, indicating which are prespecified and which are exploratory |  | Results |
| Adverse events | 19 | All important adverse events or side effects in each intervention group |  | Results |
| **Discussion** |  |  |  |  |
| Interpretation | 20 | Interpretation of the results, taking into account study hypotheses, sources of potential bias or imprecision, and the dangers associated with multiplicity of analyses and outcomes |  | Discussion |
| Generalisability | 21 | Generalisability (external validity) of the trial findings | Describe key aspects of the setting which determined the trial results. Discuss possible differences in other settings where clinical traditions, health service organisation, staffing, or resources may vary from those of the trial | Discussion |
| Overall evidence | 22 | General interpretation of the results in the context of current evidence |  | Discussion |

Extension to CONSORT checklist for noninferiority and equivalence trials

<https://pubmed.ncbi.nlm.nih.gov/23268518/>

| **Section** | **Item** | **Standard CONSORT description** | **Extension for non-inferiority trials** | **Reported in Section** |
| --- | --- | --- | --- | --- |
| Title | 1a | Identification of randomised trial in title | Identification as a non-inferiority trial in the title | Title |
| Abstract | 1b | Structured summary of trial, design, methods results and conclusions | See table below | See table below |
| **Introduction** |  |  |  |  |
| Background | 2a | Scientific background and explanation of rationale | Rationale for using a non-inferiority design | Background |
|  | 2b | Specific objectives or hypothesis | Hypothesis concerning noninferiority design, specifying the noninferiority margin with the rational for its choice | Background & Methods: sample size |
| **Methods** |  |  |  |  |
| Trial design | 3a | Description of trial design(such as parallel, factorial), including allocation ratio |  | Methods: Study design and participants  Methods: Randomisation and blinding |
|  | 3b | Important changes to methods after trial commencement (such as eligibility criteria) with reason. |  | Methods |
| Participants | 4a | Eligibility criteria for participants | Whether participants in the non-inferiority trial are similar to those in any trial(s) that established efficacy of the reference treatment | Background  Methods: Study design and participants  Methods: Randomisation and blinding  Methods: Data collection  Methods: Interventions |
|  | 4b | Settings and locations where the data were collected |  | Methods: Study design and participants  Methods: Data collection |
| Interventions | 5 | The interventions for each group with sufficient detail to allow replication, including how and when they were actually administered | Whether the reference treatment in the noninferiority trial is identical (or very similar) to that in any trial(s) that established efficacy of the reference treatment. | Background  Methods: Interventions |
| Outcomes | 6a | Completely defined prespecified primary and secondary outcome measures including how and when they were assessed | Specify the noninferiority outcome(s) and whether hypotheses for main and secondary outcome(s) are noninferiority or superiority. Whether the outcomes in the noninferiority trial are identical (or very similar) to those in any trial(s) that established efficacy of the reference treatment | Background  Methods:  Primary outcome measure  Secondary outcome measures  Sample size |
|  | 6b | Any changes to trial outcomes after the trial commenced, with reasons |  | Not applicable |
| Sample size | 7a | How sample size was determined | Whether the sample size was calculated using noninferiority criterion and, if so, what the noninferiority margin was | Methods: Sample size |
|  | 7b | When applicable explanation of any interim analyses and stopping guidelines | To which outcome(s) they apply and whether related to noninferiority hypothesis | Methods: Sample size |
| Randomisation—sequence generation | 8a | Method used to generate the random allocation sequence |  | Methods: Randomisation and blinding |
|  | 8b | Type of randomisation; details of any restriction (such as blocking and block size) |  | Methods: Randomisation and blinding |
| Randomisation—allocation concealment mechanism | 9 | Mechanism used to implement the random allocation sequence (such as sequentially numbered containers), describe any steps taken to conceal the sequence until interventions were assigned |  | Methods: Randomisation and blinding |
| Randomisation—implementation | 10 | Who generated the random allocation sequence, who enrolled participants, and who assigned participants to interventions |  | Methods: Randomisation and blinding |
| Blinding | 11a | If done, who was blinded after assignment to interventions (for example participants, care providers, those assessing outcomes) and how | If blinding was not done, or was not possible, explain why | Not applicable |
|  | 11b | If relevant, description of the similarity of interventions |  | Not applicable |
| Statistical methods | 12a | Statistical methods used to compare groups for primary and secondary outcomes | Whether a 1- or 2-sided confidence interval approach was used. | Methods: Statistical analysis |
|  | 12b | Methods for additional analyses, such as subgroup analyses and adjusted analyses |  | Not applicable |
| **Results** |  |  |  |  |
| Participant flow (a diagram is strongly recommended) | 13a | For each group, the number of participants who were randomly assigned, received intended treatment, and were analysed for the primary outcome |  | Results: Figure 1 |
|  | 13b | For each group, losses and exclusions after randomisation, together with reasons. |  | Results: Figure 1 |
| Recruitment | 14a | Dates defining the periods of recruitment and follow-up |  | Results |
|  | 14b | Why the trial ended or was stopped |  | Results |
| Baseline data | 15 | A table showing baseline demographic and clinical characteristics of each group |  | Table 1 |
| No. analysed | 16 | For each group, number of participants (denominator) included in each analysis and whether analysis was by original assigned group |  | Results, Figures 1-4, Table 1 & 2 |
| Outcomes and estimation | 17a | For each primary and secondary outcome, results for each group, the estimated effect size and its precision (such as 95% confidence interval) |  | Results |
|  | 17b | For binary outcomes, presentation of both absolute and relative effect sizes is recommended. |  | Not applicable |
| Ancillary analyses | 18 | Results of any other analysis performed, including subgroup analyses and adjusted analyses, distinguishing prespecified from exploratory |  | Results |
| Harms | 19 | All important harms or unintended effects in each group |  | Results |
| **Discussion** |  |  |  |  |
| Limitations | 20 | Trial limitations, addressing sources of potential bias, imprecision, and, if relevant, multiplicity of analysis |  | Discussion |
| Generalisability | 21 | Generalisability (external validity, applicability) of the trial findings |  | Discussion |
| Interpretation |  | Interpretation of the results, balancing benefits and harms, and considering other relevant evidence. | Interpret results in relation to noninferiority hypothesis, if a superiority conclusion is drawn for outcome(s) for which noninferiority was hypothesised, provide justification for switching | Discussion |
| Other information |  |  |  |  |
| Registration | 23 | Registration number and name of trial registry |  | Methods: Approvals |
| Protocol | 24 | Where the full trial protocol can be accessed, if available |  | Methods: Approvals |
| Funding | 25 | Sources of funding and other support (such as supply of drugs), role of funders |  | Funding and Acknowledgements |
